# A Rare Case of an Osteolytic Bone-infarct-associated Osteosarcoma: Case Report with Radiographic and Histopathologic Correlation, and Literature Review

**DOI:** 10.7759/cureus.2777

**Published:** 2018-06-11

**Authors:** Michael D McDonald, Sam Sadigh, Kristy L Weber, Ronnie Sebro

**Affiliations:** 1 Department of Anesthesiology, Perelman School of Medicine at the University of Pennsylvania, Philadelphia, USA; 2 Department of Pathology, The Hospital of the University of Pennsylvania, Philadelphia, USA; 3 Department of Orthopaedic Surgery, The Hospital of the University of Pennsylvania, Philadelphia, USA; 4 Department of Radiology, Perelman School of Medicine at the University of Pennsylvania, Philadelphia, USA

**Keywords:** bone infarct, osteosarcoma, pathological fracture, sarcoma

## Abstract

Benign lesions such as Paget’s disease of the bone, enchondroma, osteochondromas, chronic osteomyelitis/infections and bone infarcts may rarely undergo malignant degeneration/transformation into sarcomas. To date, only 14 prior bone infarct-associated osteosarcomas have been described, with just two being primarily osteolytic. We discuss a case of a patient with a humeral bone-infarct, who presented with a presumed benign pathological fracture of the humerus through the bone infarct. Subsequent imaging and biopsy showed that there was a malignant degeneration into a primarily osteolytic osteosarcoma. We review the patient’s presentation, radiographic and histologic appearance of the osteosarcoma and discuss the epidemiology, surgical and non-surgical treatment and surveillance of bone-infarct-associated osteosarcomas.

## Introduction

Bone-infarct-associated sarcomas (BIAS) were first described in 1966 by Furey et al. [1]. BIAS are thought to be the result of malignant degeneration of bone infarcts. Since these first reported cases, reports have been rare, with only a few cases described to date, and only a few published reviews analyzing BIAS. Only 14 of these cases have been osteosarcomas [2-9].

We present a case of primarily osteolytic infarct-associated osteosarcoma in a 72-year-old male patient who presented with a pathological right humeral fracture. We review his presentation, pertinent imaging, and histological findings of bone-infarct associated-osteosarcomas. In addition, we review the literature to discuss operative and non-operative treatment, and surveillance of bone-infarct-associated osteosarcomas.

## Case presentation

**Figure 1:**
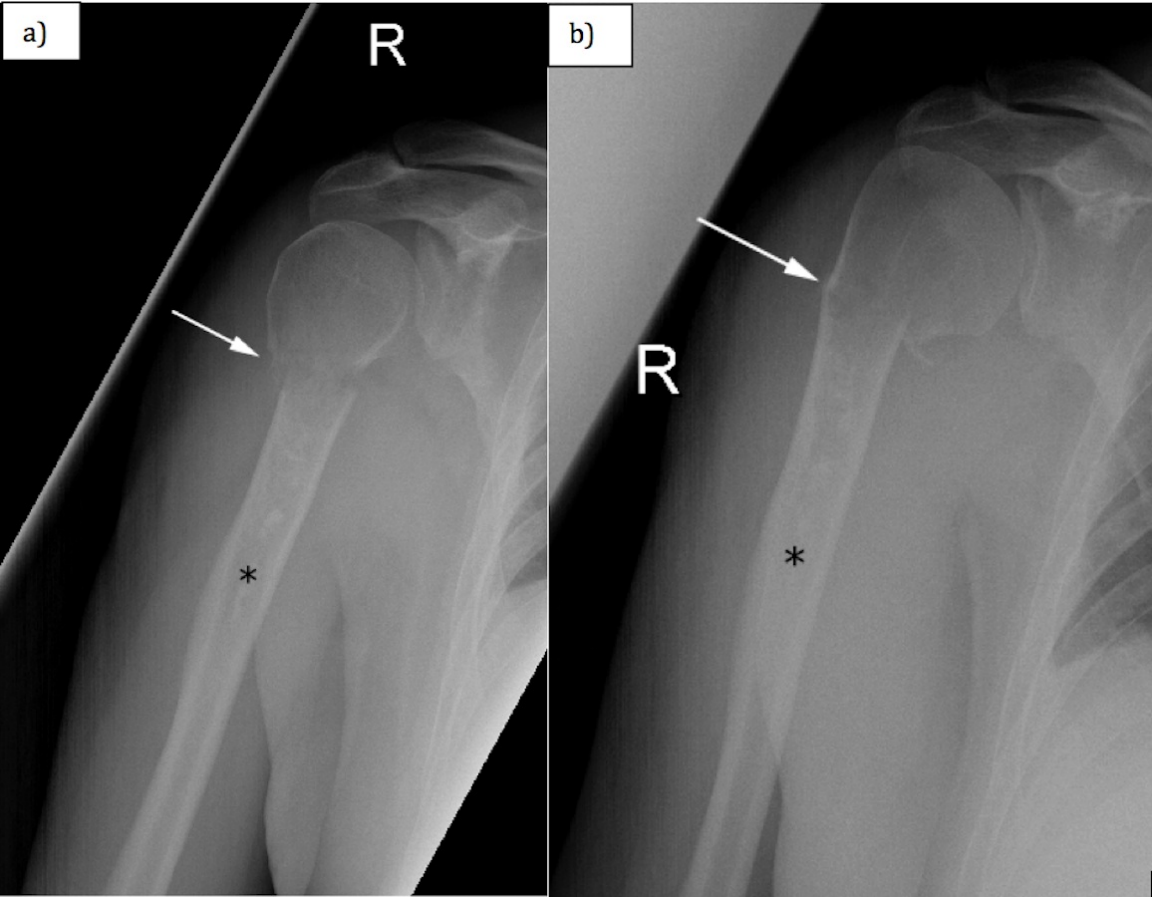
(a) Anterior-posterior and (b) slightly rotated anterior-posterior radiograph of the right humerus. White arrow shows the transverse fracture of the proximal humerus. Black asterisk shows the linear area of increased mineralization, corresponding to a bone infarct.

A 72-year-old left-handed man with past medical history of atrial fibrillation, congestive heart failure and mitral valve repair, but no history of malignancy, presented to the Emergency Department in 2016 for evaluation of right arm pain. The patient heard a crack in his arm while dressing and subsequently his arm pain worsened. His pain, when he was evaluated at the Emergency Department, was subjectively rated as 5/10, worse with activity and palpation and relieved with rest. No edema or erythema was noted. There was no axillary or cervical adenopathy. His pulses were normal and his sensation to light touch was intact. Radiographs obtained in the Emergency Department revealed a minimally angulated proximal right humeral fracture at the superior aspect of a linear sclerotic lesion in the proximal humeral diaphysis. The linear sclerotic lesion was thought to be likely a bone infarct (Figure 1a and Figure 1b). No definite soft tissue lesion was noted. His fracture was treated conservatively with splinting.

**Figure 2:**
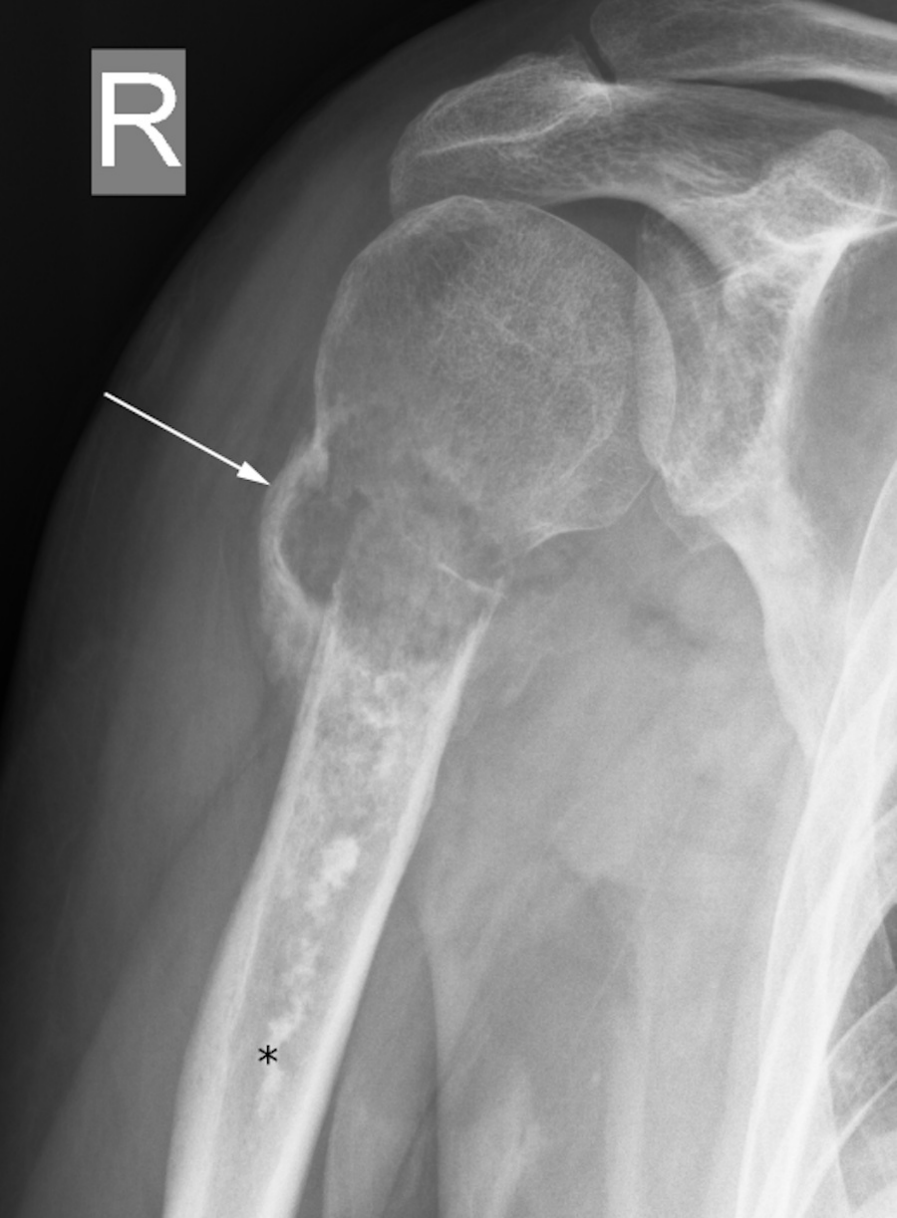
Anterior-posterior radiograph of the right shoulder. White arrow shows the transverse fracture of the proximal humerus. Black asterisk shows the linear area of increased mineralization, corresponding to a bone infarct.

**Figure 3:**
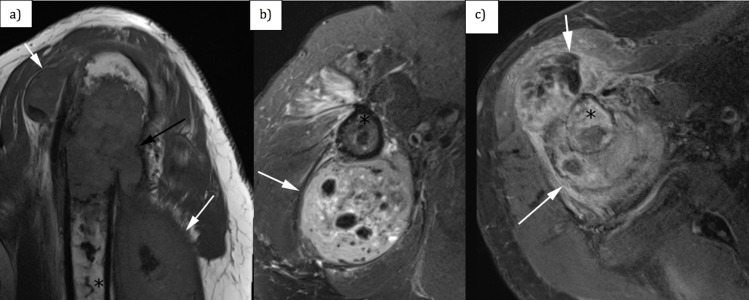
(a) Sagittal T1-weighted magnetic resonance imaging (MRI) sequence of the right shoulder (TR = 625 ms, TE = 20 ms, slice thickness 3 mm, interslice gap 0.3 mm), (b) axial T2-weighted MRI sequence of the right shoulder (TR = 4780 ms, TE = 88 ms, slice thickness 3 mm, interslice gap of 0.3 mm) and (c) axial T1-weighted MRI sequence of the right shoulder with fat saturation after administration of intravenous gadolinium contrast (TR = 649 ms, TE = 20 ms, slice thickness 3 mm, interslice gap of 0.3 mm). White arrows demarcate the soft tissue component of the lesion involving the proximal right humerus. Black asterisk shows the intramedullary component of the lesion.

However, his pain progressively worsened, so repeat radiographs were obtained a couple of weeks later to assess healing of the fracture at his fracture follow-up clinic visit (Figure 2). These subsequent radiographs demonstrated the development of a lytic lesion with surrounding periosteal reaction at the fracture site. No osteoid production/mineralization was appreciated. Magnetic resonance imaging (MRI) showed a T1-isointense (Figure 3a), T2 heterogeneously hyperintense (Figure 3b), heterogeneously enhancing lesion (Figure 3c) originating from the intramedullary cavity with osseous destruction of the humerus and a soft tissue component that measured up to 15 cm in superior-inferior dimension. A serpiginous, linear area of low T1 and low T2 signal consistent with a bone infarct was noted at the lesion, and this area of infarct extended more distally in the humeral diaphysis. This bone infarct corresponded to the linear area of sclerosis seen in the humeral diaphysis on prior radiographs.

**Figure 4:**
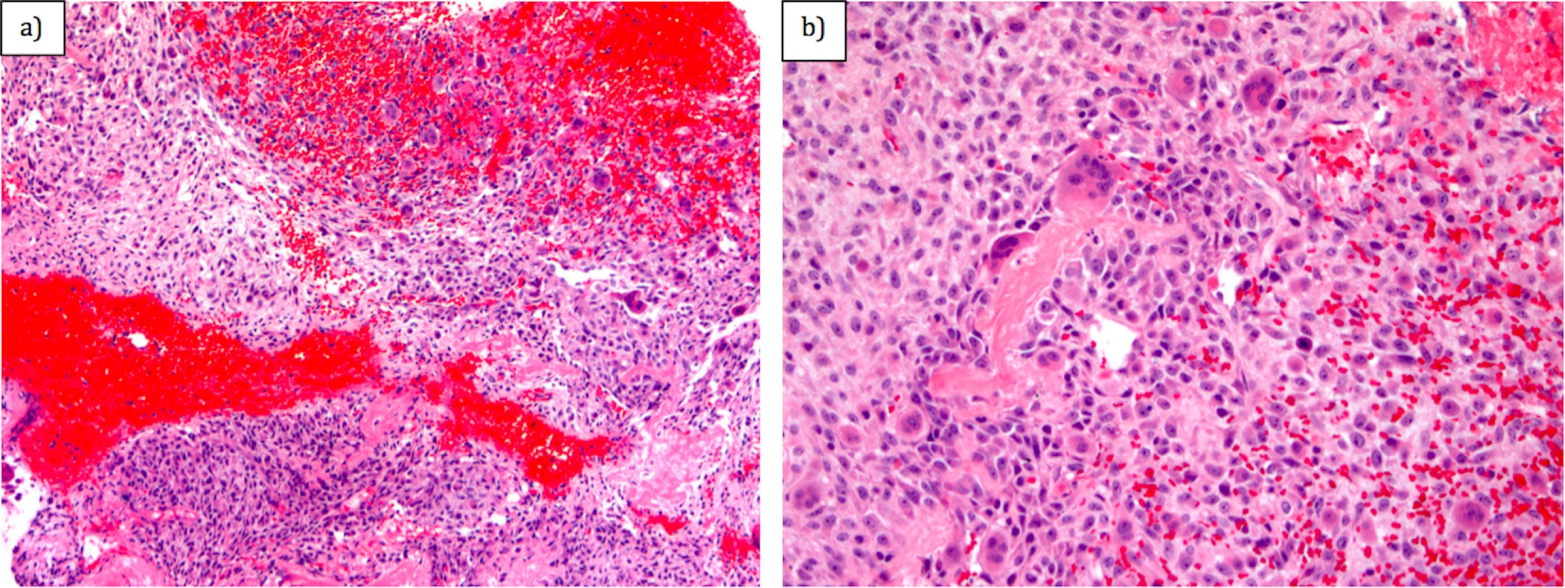
(a) Hematoxylin and Eosin (H&E) (x10) and (b) H&E (x20) analysis of biopsy showing neoplastic spindle and epithelioid cells with focal osteoid formation and a few giant cells.

**Figure 5:**
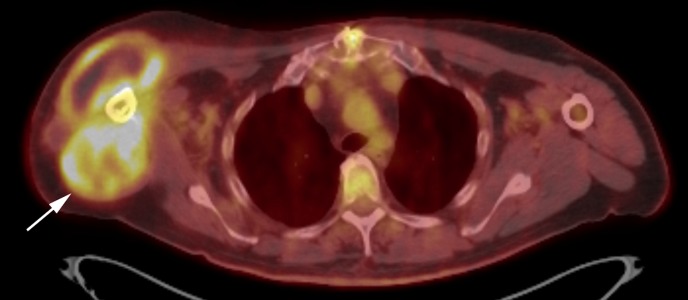
Axial fused PET/CT of the right shoulder demonstrating a hypermetabolic bone and soft tissue lesion involving the proximal right humerus. PET/CT: Positron emission tomography/computed tomography.

Ultrasound-guided core needle biopsy of the soft tissue component of the lesion was performed. Histological analysis of the biopsy showed neoplastic spindle and epithelioid cells with focal osteoid formation (Figure 4). A few giant cells were also noted. There were frequent mitotic figures (4 per high powered field). Areas of hemorrhagic necrosis were present. No chondroid material was noted. Immunohistochemical stains with adequate controls were performed. The epithelioid cells were negative for TTF-1, S100, AE1/3, PSA, HMB45, ERG, CD31, CAIX, HepPar, PAX8, and pancytokeratin. INI1 was retained. This immunoprofile combined with the histological findings supported a diagnosis of a tumor of bone origin and were most consistent with an osteogenic sarcoma (Figure 4). Staging 18F-fluorodeoxyglucose (FDG) positron emission tomography/computed tomography (PET/CT) showed localized disease (Figure 5).

He was treated with reduced dose cisplatin/adriamycin given his age. He had a poor response to chemotherapy with enlargement of the mass through chemotherapy with the development of circumferential disease around the humerus. He underwent a right forequarter amputation. He developed lung metastases and an L4 spinal metastasis with pathological fracture two months following surgery. He received palliative radiation therapy to the L4 vertebra and died seven months after initial presentation.

## Discussion

BIAS make up an estimated 1% of all bone sarcomas [2]. Most osteosarcomas are primary osteosarcomas, however secondary osteosarcomas may also arise from malignant degeneration of Paget’s disease of the bone, prior radiation, chronic osteomyelitis, prior burns, and chronic bone infarcts, like the patient described in this case [10]. Furey et al. described the first cases of BIAS in 1966 [1]. Bone infarcts are most commonly idiopathic, but can be secondary to chronic steroid use, trauma, alcohol use, sickle cell disease and other hemoglobinopathies, pancreatitis, connective tissue disease, radiation therapy, or historically Caisson disease [5, 7]. Malignant fibrous histiocytoma (now called undifferentiated pleomorphic sarcoma) (63.2%), followed by osteosarcoma (18.4%), fibrosarcoma (13.2%), anaplastic sarcoma (2.6%) and pleomorphic sarcoma (2.6%) were the histological diagnoses in a study of 38 patients with BIAS [3, 4].

To the best of our knowledge, there have only been a total of 14 bone-infarct-associated osteosarcomas reported in the literature to this date, with our case being the 15th (Table 1). The average age is 57 (range 23-79) years old. The majority (10/15) have been male. For those reporting, 6/9 (67%) have been primarily osteoblastic lesions [2-9]. Our report is the third case outlining a primarily osteolytic osteosarcoma arising at the site of prior bone infarction. Most cases presented with tumor of the femur (8/15, 53%), followed by tibia (4/15, 27%), and rarely humerus (3/15, 20%) as in the patient presented above. Of the three patients with primarily osteolytic lesions, all three died of disease progression within two years, with a mean of 11 months following diagnosis. Of the six patients with primarily osteoblastic tumors, two were lost to follow-up, one was alive and well at 120 months, and three died with a mean of 14 months following diagnosis (Table 1).

**Table 1:** All reported osteosarcomas associated with bone-infarction. LTFU: Lost to follow-up; XRT: Radiation therapy.

Case [Ref.]	Age	Sex	Location	Primarily lytic vs. blastic	Treatment	Course
1 - Torres and Kyriakos [3]	48	M	Distal tibia	Not reported	Amputation, XRT	Dead at nine months
2 - Torres and Kyriakos [3]	61	M	Distal femur	Lytic	Amputation, XRT	Dead at three months
3 - Torres and Kyriakos [3]	79	M	Femur	Blastic	Resection	Dead at 19 months
4 - Torres and Kyriakos​​​​​​​ [3]	56	F	Femur	Not reported	Amputation	Dead at 40 months
5 - Torres and Kyriakos​​​​​​​ [3]	35	M	Proximal femur	Blastic	Amputation, chemotherapy	Alive at 120 months
6 - Torres and Kyriakos​​​​​​​ [3]	61	F	Proximal tibia	Blastic	Amputation	Metastases at 12 months, LTFU
7 - Torres and Kyriakos​​​​​​​ [3]	82	M	Proximal humerus	Blastic	Amputation	Dead at 12 months
8 - Resnik et al. [8]	56	M	Proximal tibia	Not reported	Resection	Not reported
9 - Desai et al. [6]	35	F	Distal femur	Lytic	Amputation	Dead at 24 months
10 - Domson et al. [4]	61	M	Proximal tibia	Not reported	Resection, chemotherapy	Alive at 58 months
11 - Domson et al. [4]	56	F	Distal femur	Not reported	Resection	Dead at five months
12 - Domson et al. [4]	35	M	Proximal femur	Not reported	XRT	LTFU 0.5 months
13 - Bahk et al. [2]	61	M	Distal femur	Blastic	Amputation	Dead at 11 months
14 - Endo et al. [9]	56	F	Proximal humerus	Blastic	Resection	Not reported
15 - Current case	72	M	Proximal humerus	Lytic	Amputation, chemotherapy	Dead at seven months

While the pathogenesis of sarcoma development in chronic infarct is not known, some reports suggest that it involves sarcomatous change to surrounding reparative tissue [2]. Bahk et al. have described a transformation zone consisting of granulation tissue, chronic inflammatory infiltrate, and increasing cellular atypia on histology in between zones of chronically infarcted bone and sarcoma [2].

Bone-infarct-associated osteosarcomas often present with either pain or swelling in a region adjacent to a chronic bone infarct or with a pathological fracture, similar to the patient discussed [2-9]. The cause of bone infarcts is established in only one-third of patients with bone-infarct-associated osteosarcomas, with chronic steroid use and decompression sickness being implicated in previously published cases [3, 7]. In our case, there was no known etiology for the patient’s bone infarct.

Initial imaging of bone-infarct-associated osteosarcomas includes plain film radiography. Plain film radiography of bone-infarct-associated osteosarcomas demonstrates a pattern of periosteal reaction, increased bone mineralization, sometimes in association with ossification of surrounding soft tissue indicating osteoid matrix production [10]. A key point to be emphasized is that the mechanism of injury and resultant fracture in our case were atypical. The clinical history of an atraumatic fracture in conjunction with identification of a lesion that has the propensity to undergo malignant degeneration (bone infarct) should prompt further evaluation with contrast-enhanced MRI. MRI is superior to radiographs or computed tomography (CT) for evaluating lesion extent [5]. CT shows a lesion, sometimes with osteoid matrix production adjacent to a bone infarct. MRI is able to delineate in better detail the extent of soft tissue invasion and the integrity of the neurovascular structures. Osteosarcomas are typically isointense to hyperintense to skeletal muscle on T1-weighted MRI sequences. Osteosarcomas are also heterogeneously hyperintense to skeletal muscle on T2-weighted MRI sequences, and may have fluid-fluid levels or cystic change. Contrast-enhanced MRI is useful for assessing lesion extent and is useful for surgical and radiation therapy planning [5].

Staging can be done with FDG PET/CT or skeletal scintigraphy with technetium-99m methylene diphosphonate (Tc99m-MDP bone scan) [5]. FDG PET/CT is used to evaluate for metastases, although PET/CT is limited for evaluation for pulmonary nodules compared to a volumetric CT [5]. No imaging finding is reliably pathognomonic and the final diagnosis is based on histologic analysis.

Bone-infarct-associated osteosarcomas are aggressive neoplasms with poor prognosis [1-9]. Early diagnosis is key for starting treatment and surgical management prior to the development of metastases, because prognosis worsens with metastases [5]. Neoadjuvant chemotherapy is standard of care in treating osteosarcoma and used to decrease the tumor burden and increase the possibility for limb salvage [10]. Neoadjuvant chemotherapy is often utilized, and palliative chemotherapy is recommended for metastatic disease [10]. After chemotherapy, the standard of care includes wide resection of the sarcoma with limb salvage reconstruction if possible [5, 10]. A review of published cases of bone-infarct-associated osteosarcoma showed that patients who received chemotherapy in addition to surgery had a two-year survival rate of 63%, compared to a two-year survival rate of 24% in patients who received surgery alone [4]. Of the 15 reported cases, 13 included follow-up information beyond one month. Patients rapidly progressed, even following optimal treatment, with 11/13 (85%) developing with metastases, and 8/13 (62%) dying within two years (mean 11 months, range 3 to 24 months) of diagnosis. The patient presented in this case died secondary to lung and spinal metastases seven months following surgical resection. Survivors are generally followed for at least five years with serial chest CTs and radiographs to ensure no local recurrence after surgical management.

## Conclusions

Osteosarcomas can result from malignant degeneration of bone-infarcts. We present the third case of a primarily osteolytic bone-infarct-associated osteosarcoma. This lesion was locally aggressive and recalcitrant to chemotherapy with poor prognosis.
